# Assessment of mulberry leaf as a potential feed supplement for animal feeding in P.R. China

**DOI:** 10.5713/ajas.18.0671

**Published:** 2019-01-04

**Authors:** Ming Cai, Lan Mu, Zong-li Wang, Jian-yong Liu, Tian-liang Liu, Metha Wanapat, Bi-zhi Huang

**Affiliations:** 1 State Key Laboratory of Grassland Agro-ecosystems, College of Pastoral Agricultural Science and Technology, Lanzhou University, Lanzhou, Gansu 730020, China; 2 Yunnan Academy of Grassland and Animal Science, Kunming, Yunnan 650212, China; 3 China Animal Health And Epidemiology Center, Qingdao, Shandong 266032, China; 4 Tropical Feed Resources Research and Development Center, Department of Animal Science, Faculty of Agriculture, Khon Kaen University, Khon Kaen 40002, Thailand

**Keywords:** Mulberry Leaf, Sprague-Dawley Rat, Toxic Level, Protein Resource

## Abstract

**Objective:**

Mulberry (*Morus alba* L.) is a cultivated shrub grown widely in the sub-tropical and tropical areas. It has been shown that mulberry leaf contains high levels of protein while having polyphenols as phytonutrients. Therefore, it is important to conduct an experiment to assess potential toxic level from mulberry on behavior, blood hematological and coagulation parameter using Sprague-Dawley (SD) rats.

**Methods:**

Both male and female SD rats were given an intragastric administration of respective treatments of mulberry leaf intakes (control, low and high levels). Parameters of feed intake, hematological and coagulation of blood parameters, as well as liveweight changes were taken during the 7 d of adaptation, 28 d of treatment exposure, and 14 d of recovery periods, respectively. All treatment data were statistically analyzed using analysis of variance of SPSS17.0 for Windows Statistical Software following the Randomized complete block design with sex as a block.

**Results:**

Most of the parameters of the physical symptoms of the SD rats, were not significantly different (p>0.05) when compared with that of the control group. Those which remain unchanged in each dose group were, body weight (BW) gain, feed intake, the hematology and coagulation indexes. Although, there were a few individual indicators that were abnormal, but the overall physiological appearance of the rats were normal.

**Conclusion:**

Results under this experiment revealed that most hematological and coagulation parameters of the SD rats in both male and female were normal, although the weight gain of female rats in high-dose group was significantly reduced than those of the male rats. Under this study, the use of mulberry leaf up to 2 g/kg BW did not result in abnormal phenomenon in the SD rats. These findings would offer useful information for further *in vivo* feeding trials in animals to extensively use of mulberry leaf to improve animal production, particularly in P.R. China.

## INTRODUCTION

Mulberry (*Morus alba* L.) is native to northern and central China. It is cultivated in a wide range of climates because it is highly adaptable and can survive well in harsh conditions [1–3]. It is cultivated in most areas of the world, including East Asia, Southeast Asia, Europe, Africa, and America [4–6]. In fact, China has an extensive history of “mulberry sericulture cultivation”. In the past, the traditional silk industry has played an extremely important role in the development of China Silk Road and in foreign trade. However, in recent times silkworm culture in China has decreased, and a large amount of mulberry leaves remains unused. A previous report estimated that the annual output of mulberry leaves was as high as 20 tons per hectare. However, only 1% to 3% of total biomass was utilized as sericulture resource, causing a great economic loss for silkworm and mulberry farmers. Thus, there is a critical need to transform the traditional mulberry sericulture model to a new and diversified model. Previous studies have suggested that mulberry leaves are rich in nutrients especially high in crude protein content (20% of dry matter). In fact, it could be comparable to the high-quality legume alfalfa (*Medicago sativa* L.). Mulberry, in addition has good palatability and is rich in various fatty acids, minerals, vitamins, suitable bioactive substances (chlorogenic acid, benzoic acid, rutin, and astragalin) and phytonutrients (condensed tannins and saponins) [7–12]. If these mulberry resources can be processed for feeding, the pollution caused by burning could be alleviated, and it could also effectively be used as a high-quality protein forage resources, available for animal feeding in the winter and spring seasons. This could promote the healthy and sustainable development of animal husbandry in China and other countries, leading to improved economic, social, and ecological benefits. Therefore, there is a great potential for the development and utilization of mulberry leaves, as livestock and poultry feeds.

Previous studies reported that mulberry leaves were used in aquaculture, poultry, and livestock farming with beneficial outcomes [13–15]. However, in addition to a variety of nutrients, mulberry leaves also contain high level of antinutritional factors and some active substances, such as tannins, phytic acid etc. These could be potentially negative factors that could reduce the animals’ digestion of nutrients. In addition, they could combine with calcium, iron, magnesium, zinc, and other metal ions to form insoluble compounds, reducing their effectiveness. Thus, if insufficient attention is given during the feeding process, there could be an adverse effect of their utilization in animal production [16–18]. It was previously reported that adding 5% mulberry leaf powder in grass carp feed did not affect the growth performance. But when the additive amount reaches 10%, the growth performance of grass carp decreased. Mulberry leaf surface is covered with waxy layer, which is difficult to digest and use in non-ruminants. Currently, there is limited information available on the feeding safety of mulberry leaves. Further research investigation regarding antinutritional factors, the modulation of feed processing, and the systematic feeding in ruminant is still required.

Therefore, the present study investigated the effects of toxicity from mulberry leaves levels on behavior, blood hematological and coagulation parameters in male and female Sprague-Dawley (SD) rats.

## MATERIALS AND METHODS

Animal management and experimental procedures for this study was conducted under the regulations of Guide for Laboratory Animal Administration in Gansu Province (Gansu Provincial Department of Science and Technology, 2005). All efforts were made in this study to minimize the suffering of the rats.

### Experimental feeds

Fresh leaves of mulberry were harvested from Chongqing city, China. The leaves were air dried and ground into powdery form. Samples of the powdered leaf meal were mixed in to distilled water and sodium carboxymethyl cellulose (saline water) (mulberry powder 50 g: saline water 950 mL, T2; mulberry powder 100 g: saline water 900 mL, T3) and administered to the experimental rats.

### Experimental animals

Sixty SD rats (SCXK-Diank2015-002-license), male (30) and female (30), about 5 to 7 weeks of age weighting about 200 g and 175 g for male and female respectively. The experiment used a randomized complete block design (RCBD) to study both male and female SD rats, which received mulberry leaves at 1 of 3 levels. Treatments were as follows; T1 = control, T2 = 1 g/kg body weight (BW, mulberry powder 50 g: saline water 950 mL), T3 = 2 g/kg BW (mulberry powder 100 g: saline water 900 mL).

### Feeding environment and management

Animals were raised in a general animal laboratory environmental with a temperature of 20°C to 26°C and a humidity of 40% to 70%. Animals were kept in a transparent rat cage 40.5×25×20 cm (length×width×height) that had a stainless steel cage cover. The rats were fed an SPF-grade rat growth and breeding feed from the Beijing Keao Xieli Feed Co., Ltd. (Beijing, China: production license number: SCXK (Beijing) 2012-0019). Rats had *ad libitum* access to purified tap water during the experiment. Their cages contained sterilized corncob bedding that was replaced twice a week along with cage changing, cleaning, and disinfection.

### Treatments and management of the experimental rats

During testing, SD rats were acclimated for 7 days. The gavage treatment and recovery period were 28 days and 14 days, respectively.

A dose of 1 g/kg BW, and 2 g/kg BW was administered to the low and high dose respectively. In addition, there was a solvent control group. There were 10 male and 10 female rats in each treatment group. Each group was administered with respective treatment via intragastric gavage once a day for 28 days, and the gastric perfusion volume was 20 mL/kg. Rats were observed once a day for general signs of distress, and the BW and feed intake were measured once a week. At the end of the treatment period, 7males and 7 females from each group were randomly selected for haematology and blood coagulation analyzes. No additional treatments were imposed during the remaining 14 days. The rats were observed for general signs of distress once a day, and the BW was measured once a week. When the recovery period ended, the rats were collected for haematology and blood coagulation investigation.

### Statistical method and analysis

The RCBD one-way analysis of variance was used for analysis of variance using the SPSS 17.0 for Windows statistical software. When the difference was significant, multiple comparisons were performed using Duncan’s new multiple range test. The results are presented as mean±standard deviation and marked with significant levels p<0.05 indicating the statistical significances.

## RESULTS

### Chemical composition of mulberry leaf

The chemical composition of mulberry leaf is shown in Table 1 [19,20]. It is notable that mulberry leaf contains a high level of crude protein (180 to 270 g/kg), intermediate of fiber (218 to 278 g/kg neutral detergent fiber, 102 to 130 g/kg acid detergent fiber). High concentration of Ca and P were also found (5.0, 2.2 g/kg). Condensed tannins were at 0.0054 g/kg.

### General behavior changes

Rats in the low or high dose groups were administered either 1 g/kg BW or 2 g/kg BW, respectively, and the signs of possible adverse reactions were observed immediately after each administration. As soon as the gavage was administered, rats in all groups were found curling up to different extents. They also had reduced activity after each administration and gradually returned to normal after about 20 to 45 minutes. An observation of their fur, eyes, ears, mouth, nose, external genitalia, limbs, and tail revealed no abnormalities. The body temperature, secretions, and excretions were also normal. Up until the recovery period, there were no premature death cases found in any groups of SD rats. The general behavior of all rats was normal, and the spontaneous activity was remained normal. The skin coat was clean, and no toxic symptoms were observed.

### Effect of mulberry leaves on the body weight of Sprague-Dawley rats

**Figure 1:**
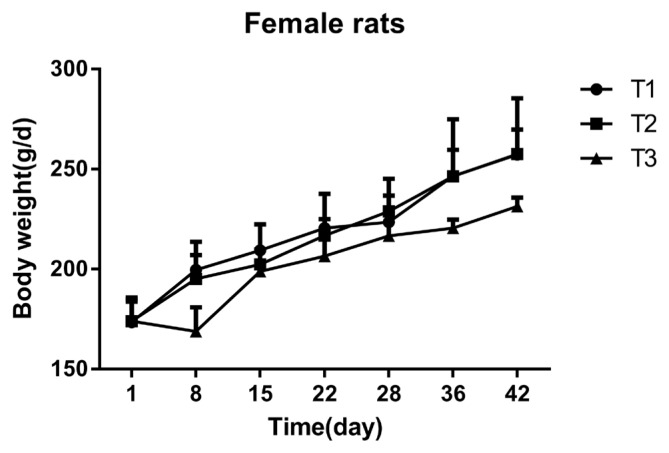
Effect of mulberry leaves on the body weight of Female rats. The x- and y-axes indicate time and body weight, respectively. T1 = control group; T2 = low dose group; T3 = high dose group. Error bars are standard error of the mean.

**Figure 2:**
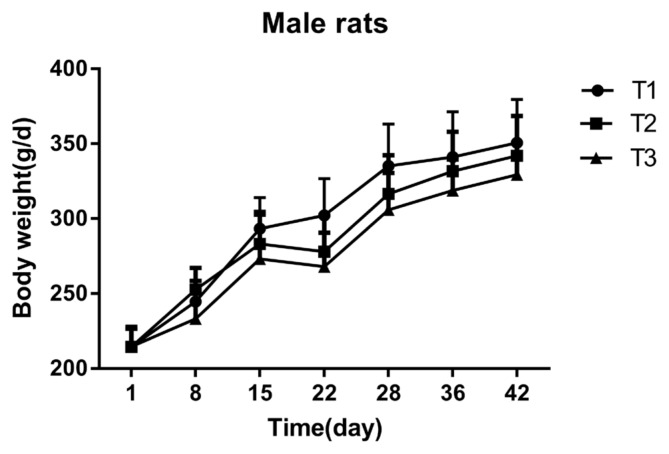
Effect of mulberry leaves on the body weight of male rats. The x- and y-axes indicate time and body weight, respectively. T1 = control group; T2 = low dose group; T3 = high dose group. Error bars are standard error of the mean.

As shown in (Figures 1, 2), the overall BW of each test group at the end of the gavage and the recovery period increased with time. In the early stage of the experiment, rats administered mulberry leaves had a significant reduction in BW, but the effect was gradually eliminated as the gavage time continued. Female rats in the high dose treatment-group had a significant reduction (p<0.05) in BW on the 8th day of treatment, when compared to both the low dose and control groups. Additionally, the BW of female rats in the high dose group was significantly decreased (p<0.05) on the 15th and 22nd day of treatment, compared to the control group. On the 28th day of treatment, there was no significant difference found among the females in the high-dose, low-dose, and control groups. The BW of male rats in the high dose group was significantly reduced on the 22nd and 28th day of treatment (p<0.05), when compared to the low dose and the control groups. However, there was no difference in BW between the groups during the recovery period.

### Effect of mulberry leaves on feed intake of Sprague-Dawley rats

**Figure 3:**
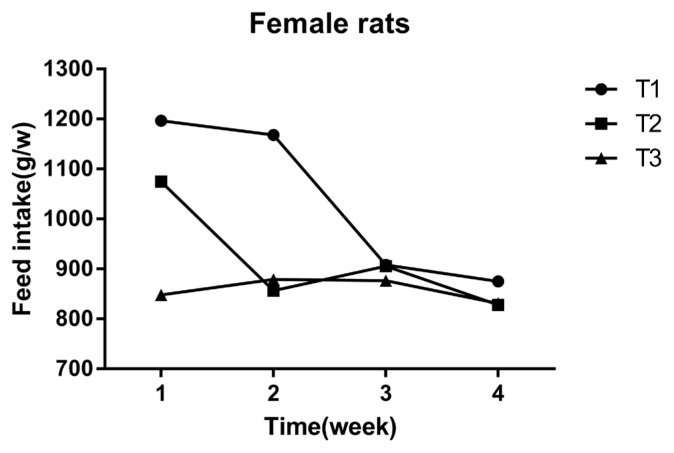
Effect of mulberry leaves on feed intake of Female rats. The x- and y-axes indicate time and feed intake, respectively. T1 = control group, T2 = low dose group, T3 = high dose group.

**Figure 4:**
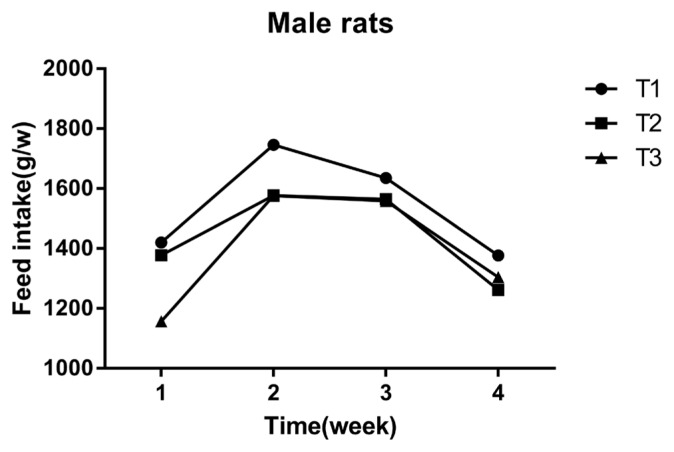
Effect of mulberry leaves on feed intake of Male rats. The x- and y-axes indicate time and feed intake, respectively. T1 = control group, T2 = low dose group, T3 = high dose group.

The feed intake was measured weekly by consumption per cage of 10 rats. It was calculated as the difference between the amount of feed given and the amount of feed remaining at the end of the week. The feed intake per week of each group of rats was recorded as only one numerical value, so the feed intake data was just used as descriptive analysis. As shown in (Figures 3, 4) the final feed intake measurement at the 4th week in the high and low dose groups was similar to the control group. The feed intake of female rats at the 1st and 2nd weeks, were lower than that of the control group, and at the 3rd and 4th weeks it was similar to the control group. Male rats in the high dose group had slightly lower BW on the 4th week, when compared to the control group.

### Effect of mulberry leaves on haematological parameters of Sprague-Dawley rats at the end of the treatment period

Statistical analysis was conducted on female and male rats separately, as well as total rats (male and female combined). High and low dose mulberry groups were compared to the control group in the same period to determine changes in hematological parameters at the end of the treatment period (Table 2). There was significant decrease (p<0.05) in mean corpuscular volume (MCV) levels in male rats in the high dose group. MCV and mean corpuscular hemoglobin (MCH) levels decreased in males, in the low dose group, which was significantly different from the control group (p<0.05). For female rats, there was no significant difference found in the various parameters. Overall, there were no differences found in various hematological parameters.

### Effect of mulberry leaves on haematological parameters of Sprague-Dawley rats at the end of recovery period

Statistical analysis was conducted on all rats (male and female combined). Groups treated with the high and low doses of mulberry leaves were compared to the control group in the recovery period to determine changes in hematological parameters (Table 3). In the low dose group, red blood cell count and hemoglobin were significantly increased (p<0.05), while red cell distribution width was decreased (p<0.05). There were no significant differences found in other remaining parameters. (p>0.05).

### Effect of mulberry leaves on coagulation parameters of Sprague-Dawley rats at the end of the treatment period

Statistical analysis was conducted on female and male SD rats separately, as well as in total rats. The groups treated with the high and low doses of mulberry leaves were compared to the control group during the same period to determine the changes in hematological parameters at the end of the treatment period (Table 4). There was a significant increase in thrombin time (TT) found in the females of the high dose group, as well as in the total rats (p<0.05). The remaining coagulation parameters were not significantly different (p>0.05), which indicates that there was no toxicological symptoms.

### Effect of mulberry leaves on coagulation parameters of Sprague-Dawley rats at the end of the recovery period

Statistical analysis was conducted on all rats (males and females combined). Groups treated with the high and low doses of mulberry leaves were compared to the control group in the recovery period to determine changes in coagulation parameters of rats at the end of the recovery period (Table 5). There were no significant differences in the coagulation parameters found in the treated groups as compared to the solvent control group (p>0.05), which indicates that there was no adverse effect.

## DISCUSSION

Mulberry leaf contains high crude protein and condensed tannins. The presence of condensed tannins could be useful in enhancing rumen fermentation [21]. Under this study, the condensed tannins were relatively higher than those found in other reports [21].

With the advancement of human society and the rapid development of agriculture and animal husbandry, the demand for meat, milk, eggs, and poultry food products for human consumption have been rising, and thus, food safety and security awareness is increasing. There is often a shortage of high-quality protein feed and an insufficient supply of green forage during the winter and spring feeding season. This greatly limits the output of livestock and poultry food products, and it threatens the safety and security of livestock and poultry food production. Livestock and poultry are high-quality protein foods for humans. Therefore, the quality of livestock and poultry feed ingredients is closely related to human health. Mulberry leaves are rich in nutrients especially with a high crude protein content, and they provide balanced amino acids with high digestibility [22,23]. In addition to being rich in nutrients, mulberry leaves also contain bioactive substances, such as jasmonic acid, anthocyanins, flavonoids, stilbene, and terpenoids [24,25]. These bioactive substances have positive effects, such as antibacterial, antipyretic, anticancer, antioxidation, hypoglycemic, serum lipid-lowering, and metabolism-improving properties [26,27]. However, they could also influence the physiology of animals. Currently, there is limited research on the safety of the mulberry leaf if used as an animal feed. As there are large mulberry plantations in China, the quantity and quality of mulberry leaves could make it an advantageous feed resource. However, the safety and toxicological evaluation of mulberry needs to be examined before it can be promoted as a feed for livestock production.

In the current study, SD rats were orally administered mulberry leaves for 28 days, and a subacute toxicity test was conducted after 14 days of a recovery period. Throughout the experiment, the physical condition, BW, and feed intake were recorded. Blood was sampled at the end of the gavage and recovery periods for analyses of hematology and coagulation parameters to evaluate the safety use of mulberry leaves. The experiment complied with the Chinese Food Standard for the 28-day Oral Toxicity Test of National Food Safety Standard (GB 15193.22-2014). The results indicated that mulberry leaves can be considered safe. There were no adverse effects on general behavior, BW, hematology, and coagulation parameters obtained in the SD rats tested.

These parameters are important indicators of the relationship between the dosage given and symptoms of toxicity [28]. The general behavior of the rats treated with the low or high doses of mulberry leaves solution and those treated with the solvent control groups were normal. All SD rats performed well on the spontaneous activity test, and their skin coat was kept clean during the gavage and recovery periods. Furthermore, there were no cases of death, and no toxic symptoms appeared. After the treatment was administered, some rats were found curling up. This symptom could be related to the concentration of the mulberry leaves treatment solution. The maximum dose of mulberry leaves was a thick suspension with a large concentration, and the oral administration volume was 20 mL/kg BW, which is a large intragastric volume for rats. After intragastric administration, the limit of gastric capacity was rapidly reached, and thus, this symptom was not due to mulberry leaf toxicity. There were no abnormal cases found in any of the experimental groups. This finding was consistent with the behavioral performance of other animals at the beginning of the treatment.

The BW and feed intake are the most sensitive indicators of toxic effects caused by a test substance. Compared with the control group, the BW of SD rats in the mulberry leaf-treated groups was significantly decreased during the initial gavage administration. This could be related to the discomfort of the initial gavage, and as the gavage period continued, the reduction in BW was gradually eliminated. A decrease in feed intake was observed, which could be due to the fact that a large volume of the test substance occupied the stomach capacity. This could reduce the feed intake, rather than a toxicological effect of the test substance itself. In general, the feed intake in the low and high dose groups at 4 weeks was lower than in the control group. A previous study indicated that the alkaloids in mulberry leaves could bind to α-glycosidase. Which could reduce the decomposition of disaccharides. In addition, the flavonoids in mulberry leaves have antioxidant activity, which promotes cells to restore insulin levels. Insulin secretion is increased, and the oxidative degradation of sugars is accelerated. This causes an impact on BW and feed intake. Mulberry leaves are capable of creating the optimum condition for rumen fermentation [29]. In the late stage of the treatment and during the recovery period, the levels of the indicators returned to normal.

Blood plays an important role in maintaining normal metabolism in animals and maintaining a balance with the external environment. Any stimulus could affect an organism by causing changes in blood components, so blood tests are one of the most common and important tests in the diagnosis of animal diseases. Compared with the control group, the haematology and coagulation parameters of male SD rats in both the low and high dose groups were increased. The low doses group had reduced levels of MCV and MCH. However, these were slight changes, indicating that it was due to normal variability and not toxic symptoms.

For the coagulation index, prothrombin time, activated partial thromboplastin time, fibrinogen, and TT were measured to determine if the rats had haemorrhagic symptoms. Female SD rats in the high dose group and in the total group demonstrated an increase in TT (p<0.05). The detection of TT indicates that the clotting time is prolonged. This should result in an increase in heparin and hepatic substances, but the other coagulation parameters were not significantly changed (p> 0.05). Thus, we conclude that these changes had no clear toxicological significance because many indicators of haematology were mutually influenced, rather than independently. Since a single indicator was lacking on biological significance, it suggests that the change had no clear toxicological significance.

## CONCLUSION

Under this experiment, it could be concluded that feeding the experimental rats at 2.00 g/kg BW, did not reveal significant adverse effects. This experiment preliminarily verifies the safety of mulberry leaves for use as animal feed, which provides a foundation for further research and exploitation of mulberry leaves as a livestock feed. Nevertheless, *in vivo* feeding trials need to be conducted to evaluate more practical implications for the extensive use of mulberry leaves in animal production, particularly in P.R. China.

## Figures and Tables

**Table 1:** Chemical composition of mulberry leaf

Items	g/kg DM
DM	180–270
Ash	108–128
CP	170–194
NDF	218–278
ADF	102–130
Lignin	15–29
Ca	5.0
P	2.2
Condensed tannins	0.0054
r-Aminobutyric acid	0.0021

DM, dry matter; CP, crude protein; NDF, neutral detergent fiber; ADF, acid detergent fiber.

**Table 2:** Effect of mulberry leaves on hematological parameters of Sprague-Dawley rats at the end of the treatment period

Items	T11)			T21)			T31)		
		
Gender	Female	Male	Total	Female	Male	Total	Female	Male	Total
WBC (10^9^/L)	5.86±1.4	8.39±2.27	7.12±2.24	6.40±2.02	8.97±5.17	7.69±4.00	7.33±2.63	5.50±2.40	6.41±2.59
Lymph (10^9^/L)	4.21±1.03	6.01±1.73	5.11±1.66	4.64±1.51	5.77±2.66	5.21±2.16	5.27±1.90	3.73±1.68	4.50±1.90
Mon (10^9^/L)	0.56±0.35	0.69±0.33	0.62±0.34	0.53±0.22	0.67±0.47	0.60±0.36	0.56±0.33	0.40±0.34	0.48±0.33
Gran (10^9^/L)	1.09±0.22	1.69±0.57	1.39±0.52	1.23±0.49	2.53±2.39	1.88±1.79	1.50±1.05	1.37±0.61	1.44±0.83
Lymph (mg/dL)	71.54±4.20	71.47±4.80	71.51±4.33	72.24±4.57	66.11±8.43	69.18±7.25	71.89±10.69	66.66±5.17	69.27±8.51
Mon (mg/dL)	9.93±3.60	8.61±4.18	9.27±3.81	8.23±3.82	8.29±4.06	8.26±3.79	8.37±4.83	7.34±4.13	7.86±4.35
Gran (mg/dL)	18.53±3.27	19.91±2.31	19.22±2.82	19.53±3.62	25.60±7.74	22.56±6.60	19.74±7.37	26.00±5.10	22.87±6.90
RBC (10^12^/L)	8.27±0.58	9.02±0.64	8.65±0.70	8.51±0.77	8.59±2.24	8.55±1.61	8.23±0.38	8.40±1.48	8.32±1.04
HCT (mg/dL)	46.99±2.98	51.71±3.94	49.35±4.16	49.40±6.18	47.13±12.71	48.26±9.68	46.99±2.59	46.90±8.58	46.94±6.09
MCV (fL)	56.90±1.68^a^	57.34±0.84^a^	57.12±1.30^a^	57.97±3.78^a^	54.79±1.51^bc^	56.38±3.22^a^	57.11±1.80^a^	55.84±0.86^b^	56.48±1.51^a^
MCH (pg)	18.11±0.52^a^	18.23±0.60^a^	18.17±0.54^a^	18.33±0.84^a^	17.44±0.45^b^	17.89±0.79^a^	17.90±0.63^a^	17.69±0.45^a^	17.79±0.54^a^
MCHC (g/L)	319.29±8.28	318.57±8.70	318.93±8.17	317.43±8.14	319.00±5.69	318.21±6.80	314.29±7.61	317.29±6.80	315.79±7.11
RDW (mg/dL)	12.70±1.44	13.06±1.52	12.88±1.43	12.31±1.82	11.79±0.52	12.05±1.32	13.19±1.25	13.43±1.20	13.31±1.18
MPV (fL)	6.07±0.44	6.13±0.31	6.10±0.37	5.90±0.57	6.01±0.18	5.96±0.41	6.11±0.35	6.16±0.48	6.14±0.40
PDW (mg/dL)	16.10±0.45	16.01±0.28	16.06±0.36	16.06±0.05	16.17±0.65	16.11±0.45	15.90±0.08	16.10±0.71	16.00±0.50
HGB (g/L)	150.29±11.27	164.86±11.47	157.57±13.28	156.71±17.05	150.86±41.09	153.79±30.38	147.71±6.99	149.14±27.41	148.43±19.23

WBC, White blood cell count; Lymph, lymphocytes; Mon, monocytes; Gran, granulocyte; RBC, red blood cell count; HCT, hematocrit; MCV, mean corpuscular volume; MCH, mean corpuscular hemoglobin; MCHC, mean corpuscular-hemoglobin concentration; RDW, red cell distribution width; MPV, mean platelet volume; PDW, platelet distribution width; HGB, hemoglobin.

1) T1 = control group; T2, low dose group; T3, high dose group.

The results are presented as mean±standard deviation and marked with significant levels.

a–c With different superscripts in the same row indicate significant differences (p<0.05).

**Table 3:** Effect of mulberry leaves on hematological parameters of Sprague-Dawley rats at the end of recovery period

Items	T11)	T21)	T31)
WBC (10^9^/L)	7.78±2.33	7.48±6.33	8.42±2.74
Lymph (10^9^/L)	4.87±1.51	4.42±2.83	5.73±1.64
Mon (10^9^/L)	0.80±0.44	0.58±0.48	0.62±0.25
Gran (10^9^/L)	2.12±0.86	2.48±3.13	2.07±1.14
Lymph (mg/dL)	62.52±6.77	64.22±8.43	68.65±4.40
Mon (mg/dL)	10.78±4.62	8.40±2.60	7.27±2.28
Gran (mg/dL)	26.70±7.67	27.38±9.16	24.08±6.30
RBC (10^12^/L)	8.06±0.57^a^	8.72±0.44^b^	8.49±0.55^a^
HCT (mg/dL)	44.85±3.31	48.60±3.25	46.72±3.67
MCV (fL)	55.72±1.83	55.87±2.48	55.07±2.23
MCH (pg)	17.80±0.76	18.10±1.00	17.87±0.64
MCHC (g/L)	320.00±6.99	324.83±6.79	325.00±2.10
RDW (mg/dL)	14.27±1.22^a^	12.75±1.00^b^	14.00±1.11^a^
MPV (fL)	5.78±0.26	5.93±0.28	5.98±0.39
PDW (mg/dL)	15.83±0.08	15.85±0.14	15.88±0.10
HGB (g/L)	143.67±9.63^a^	158.17±12.61^b^	152.00±11.05^a^

WBC, White blood cell count; Lymph, lymphocytes; Mon, monocytes; Gran, granulocyte; RBC, red blood cell count; HCT, hematocrit; MCV, mean corpuscular volume; MCH, mean corpuscular hemoglobin; MCHC, mean corpuscular-hemoglobin concentration; RDW, red cell distribution width; MPV, mean platelet volume; PDW, platelet distribution width; HGB, hemoglobin.

1) T1, control group; T2 = low dose group; T3, high dose group.

The results are presented as mean±standard deviation and marked with significant levels.

a,b With different superscripts in the same row indicate significant differences (p<0.05).

**Table 4:** Effect of mulberry leaves on coagulation parameters of Sprague-Dawley rats at the end of the treatment period

Items	T11)			T21)			T31)		
Gender	Female	Male	Total	Female	Male	Total	Female	Male	Total
PT(s)	14.95±2.55	16.19±3.82	15.57±3.18	14.08±2.60	16.63±2.51	15.35±2.79	16.71±4.59	16.76±3.11	16.74±3.77
APTT(s)	46.29±9.47	52.50±13.96	49.40±11.90	56.03±14.75	57.95±21.41	56.99±17.69	52.15±7.20	48.46±6.34	50.31±6.79
TT(s)	16.12±7.24^a^	16.77±6.49^a^	16.45±6.61^a^	18.74±5.37^a^	23.67±5.05^a^	21.21±5.63^a^	24.22±6.97^bc^	21.92±8.68^a^	23.07±7.66^b^
FIB(s)	9.78±1.10	8.92±1.61	9.35±1.40	8.86±1.67	8.33±1.14	8.59±1.40	9.82±1.68	9.51±2.83	9.67±2.24

PT, prothrombin time; APTT, activated partial thromboplastin time; TT, thrombin time; FIB, fibrinogen.

1) T1 = control group; T2, low dose group; T3, high dose group.

The results are presented as mean±standard deviation and marked with significant levels.

a–c With different superscripts in the same row indicate significant differences (p<0.05).

**Table 5:** Effect of mulberry leaves on coagulation parameters of Sprague-Dawley rats at the end of the recovery period

Items	T11)	T21)	T31)
PT	14.82±1.06	14.79±2.75	13.38±3.13
APTT	33.73±10.55	37.78±13.16	45.59±7.43
TT	19.25±5.12	18.80±9.86	16.59±6.25
FIB	7.98±2.10	8.89±1.83	8.69±3.29

PT, prothrombin time; APTT, activated partial thromboplastin time; TT, thrombin time, FIB, fibrinogen.

1) T1, control group; T2, low dose group; T3, high dose group.

The results are presented as mean±standard deviation and marked with significant levels.
